# Efficacy, Usability, and Acceptability of a Chatbot for Promoting COVID-19 Vaccination in Unvaccinated or Booster-Hesitant Young Adults: Pre-Post Pilot Study

**DOI:** 10.2196/39063

**Published:** 2022-10-04

**Authors:** Tzu Tsun Luk, Judy Hiu Tung Lui, Man Ping Wang

**Affiliations:** 1 School of Nursing The University of Hong Kong Pokfulam China (Hong Kong)

**Keywords:** COVID-19, coronavirus, vaccine, immunization, booster, vaccine hesitancy, chatbot, conversational agent, virtual assistant, Chinese, young adult, youth, health promotion, health intervention, chatbot usability, pandemic, booster hesitancy, web-based survey, students, university students

## Abstract

**Background:**

COVID-19 vaccines are highly effective in preventing severe disease and death but are underused. Interventions to address COVID-19 vaccine hesitancy are paramount to reducing the burden of COVID-19.

**Objective:**

We aimed to evaluate the preliminary efficacy, usability, and acceptability of a chatbot for promoting COVID-19 vaccination and examine the factors associated with COVID-19 vaccine hesitancy.

**Methods:**

In November 2021, we conducted a pre-post pilot study to evaluate “Vac Chat, Fact Check,” a web-based chatbot for promoting COVID-19 vaccination. We conducted a web-based survey (N=290) on COVID-19 vaccination at a university in Hong Kong. A subset of 46 participants who were either unvaccinated (n=22) or were vaccinated but hesitant to receive boosters (n=24) were selected and given access to the chatbot for a 7-day trial period. The chatbot provided information about COVID-19 vaccination (eg, efficacy and common side effects), debunked common myths about the vaccine, and included a decision aid for selecting vaccine platforms (inactivated and mRNA vaccines). The main efficacy outcome was changes in the COVID-19 Vaccine Hesitancy Scale (VHS) score (range 9-45) from preintervention (web-based survey) to postintervention (immediately posttrial). Other efficacy outcomes included changes in intention to vaccinate or receive boosters and willingness to encourage others to vaccinate on a scale from 1 (not at all) to 5 (very). Usability was assessed by the System Usability Scale (range 0-100). Linear regression was used to examine the factors associated with COVID-19 VHS scores in all survey respondents.

**Results:**

The mean (SD) age of all survey respondents was 21.4 (6.3) years, and 61% (177/290) of respondents were female. Higher eHealth literacy (B=–0.26; *P*<.001) and perceived danger of COVID-19 (B=–0.17; *P*=.009) were associated with lower COVID-19 vaccine hesitancy, adjusting for age, sex, chronic disease status, previous flu vaccination, and perceived susceptibility to COVID-19. The main efficacy outcome of COVID-19 VHS score significantly decreased from 28.6 (preintervention) to 24.5 (postintervention), with a mean difference of –4.2 (*P*<.001) and an effect size (Cohen *d*) of 0.94. The intention to vaccinate increased from 3.0 to 3.9 (*P*<.001) in unvaccinated participants, whereas the intention to receive boosters increased from 1.9 to 2.8 (*P*<.001) in booster-hesitant participants. Willingness to encourage others to vaccinate increased from 2.7 to 3.0 (*P*=.04). At postintervention, the median (IQR) System Usability Scale score was 72.5 (65-77.5), whereas the median (IQR) recommendation score was 7 (6-8) on a scale from 0 to 10. In a post hoc 4-month follow-up, 82% (18/22) of initially unvaccinated participants reported having received the COVID-19 vaccine, whereas 29% (7/24) of booster-hesitant participants received boosters.

**Conclusions:**

This pilot study provided initial evidence to support the efficacy, usability, and acceptability of a chatbot for promoting COVID-19 vaccination in young adults who were unvaccinated or booster-hesitant.

## Introduction

COVID-19 vaccines are highly effective in preventing severe disease and death but are underused. By mid-2022, the full vaccination rate has remained suboptimal in many places where COVID-19 vaccines are readily available (eg, 67% in the United States and 75% in the United Kingdom) [1]. COVID-19 booster shots are also being delivered to address waning immunity and viral variants, but studies have shown that some fully vaccinated people were unwilling to take the booster [2-4]. COVID-19 may also become an endemic disease such as seasonal influenza, and regular vaccination may be needed to protect high-risk populations. Interventions to promote the vaccine is crucial to reduce the burden of COVID-19.

Vaccine hesitancy is considered 1 of the 10 major threats of global health according to the World Health Organization (WHO) [5]. Studies have consistently shown higher COVID-19 vaccine hesitancy in women, younger people, ethnic minority populations, and people with lower socioeconomic status [6,7]. Partly due to the fast-tracked development and authorization of the vaccines, the lack of confidence in the vaccine efficacy and safety were among the main drivers for hesitancy [7]. Widespread misinformation against the vaccine further amplified its safety concerns [8,9]. Debunking such misinformation could reduce COVID-19 vaccine hesitancy and promote uptake, especially in subpopulations that are more susceptible to misinformation such as young people [10].

Chatbots or conversational agents are increasingly developed as a scalable and accessible platform for supporting health care delivery. The interface of a chatbot that is familiar to most people with experiences in mobile messaging could promote the usability and user engagement of the chatbot compared to other platforms. Several chatbots have been developed amid the COVID-19 pandemic [11,12], mostly for symptom checking and information dissemination [13,14]. The WHO has also launched chatbots on popular social networking sites such as WhatsApp to provide instant and credible information about COVID-19, including vaccination [15]. Nevertheless, empirical evidence on the utility of chatbots for promoting vaccination has remained scarce.

Mass COVID-19 immunization has begun in February 2021 in Hong Kong. Despite ample supply of both an inactivated vaccine (CoronaVac; Sinovac) and mRNA vaccine (Comirnaty; Fosun-BioNTech), the uptake had been slowed, with only 62% of the population being fully vaccinated by the beginning of 2022 [1]. We conducted a population-based survey on 1501 general adults in Hong Kong (COVID-19 Health Information Survey) and found higher COVID-19 vaccine hesitancy among young adults (aged 18-29 years) than older adults (aged ≥30 years) [16,17]. We also found the low perceived COVID-19 severity and safety concerns of the vaccines to be the main drivers of vaccine hesitancy [16]. Additionally, the COVID-19 Health Information Survey showed that eHealth literacy was associated with adherence to mask wearing, hand washing and social distancing [17], but its role in vaccine hesitancy has remained under-studied. Therefore, the primary aim of the study was to examine the preliminary efficacy, usability, and acceptability of using a chatbot for promoting COVID-19 vaccination. We also examined the feasibility of assessing the long-term effect on COVID-19 vaccination status in a post-hoc 4-month follow-up. The secondary aim was to examine the factors associated with COVID-19 vaccine hesitancy, including eHealth literacy.

## Methods

### Study Design and Recruitment

We conducted a pilot study using a pretest-posttest design to evaluate “Vac Chat, Fact Check,” a chatbot designed to provide updated information and debunk misinformation about the COVID-19 vaccine. The study was conducted in November 2021, between the end of the fourth wave (June 2021) and the start of the fifth wave (January 2022) of the outbreak in Hong Kong, which had about 12,000 cumulative cases and 200 deaths.

**Figure 1:**
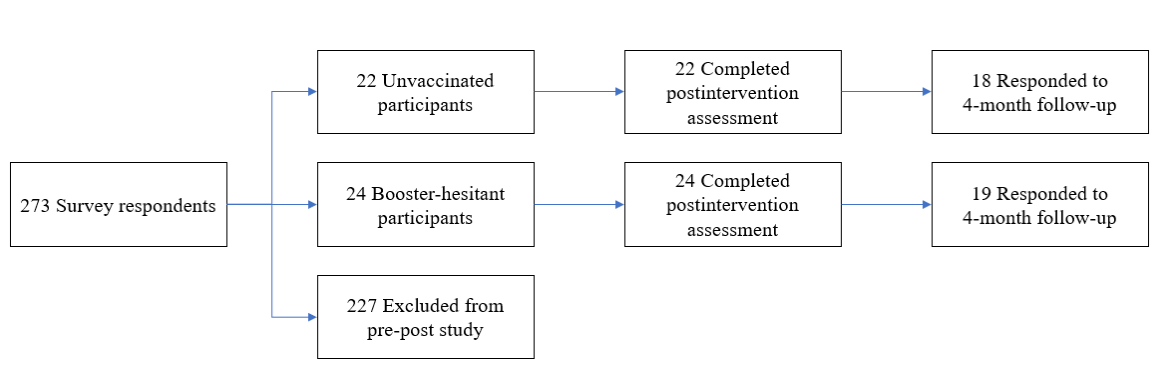
Study flow diagram.

The study targeted adults aged ≥18 years who can read and communicate in Chinese. A mass email with a link to a web-based survey of COVID-19 vaccination was sent to all students at a public university in Hong Kong on November 8, 2021. The survey link was open for 7 days and received 290 valid responses. Of these, 273 (94.1%) respondents indicated their interest in participating in the pre-post evaluation of the chatbot by leaving their contact information at the end of the survey. We identified and invited all 46 respondents who were either unvaccinated (n=22) or fully vaccinated but hesitant to receive boosters if eligible (n=24; response rate: 46/46, 100%). The planned sample size (20-25 each for unvaccinated or booster-hesitant participants) was based on a previous formative study of a chatbot for promoting human papillomavirus vaccination [18]. Figure 1 shows the study flow diagram.

### Ethics Approval

The study was approved by the Institutional Review Board of the University of Hong Kong/Hospital Authority Hong Kong West Cluster (UW 21-449).

### Study Procedures

Participants who were invited to participate in the pre-post study received a WhatsApp message describing the study purpose and provided informed consent. The participants then received a URL link to access the web-based chatbot and start a 7-day trial period. The chatbot could be accessed repeatedly. WhatsApp reminders to use the chatbot were sent on day 3, day 5, and day 7. On day 8, we sent a URL link to the postintervention questionnaire. Participants who completed the pre-post study were given HK $300 (US $38.5) for their time and effort.

On March 30, 2022, about 4 months after the completion of the pre-post study, we conducted a post hoc follow-up with a single question about COVID-19 vaccination or booster status via WhatsApp. Participation was voluntary, and consent was obtained from those who responded to the question. The additional follow-up served to examine the feasibility of measuring the long-term effect of the chatbot.

### Design of the Chatbot

**Figure 2:**
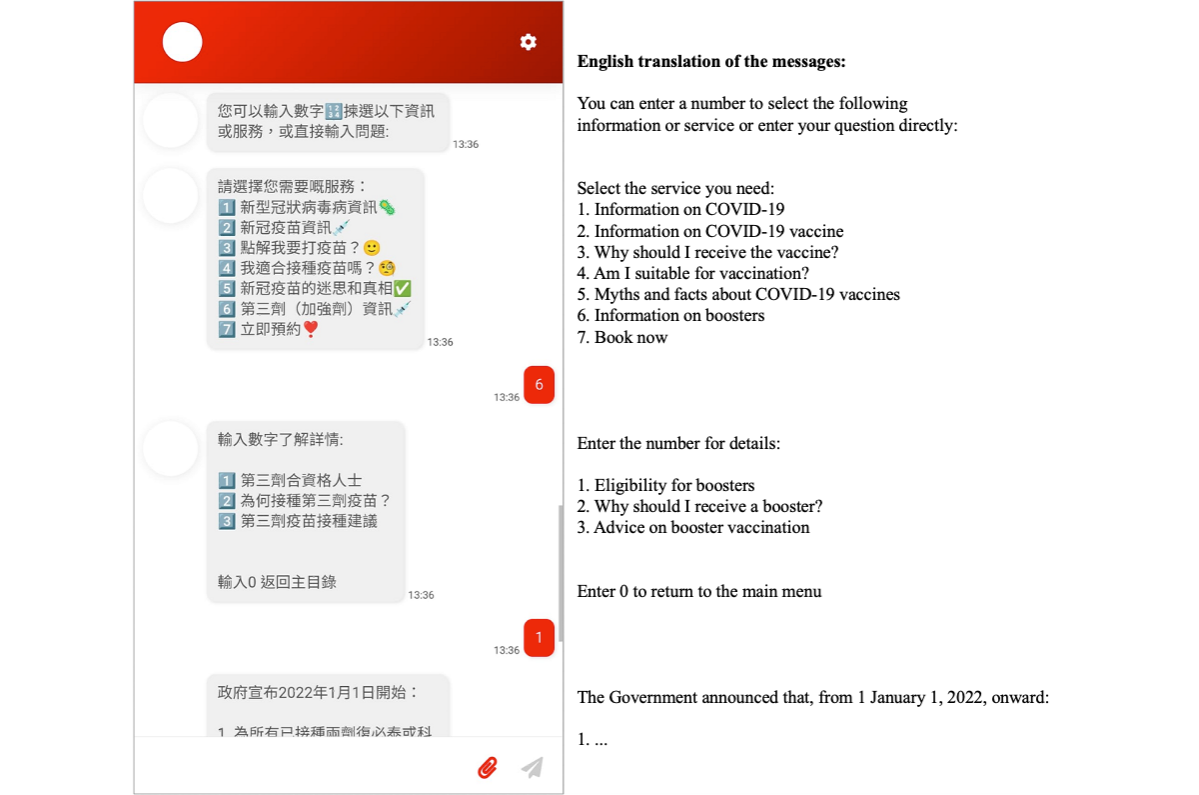
Screenshot of “Vac Chat, Fact Check” showing chatbot navigation by menu options.

**Figure 3:**
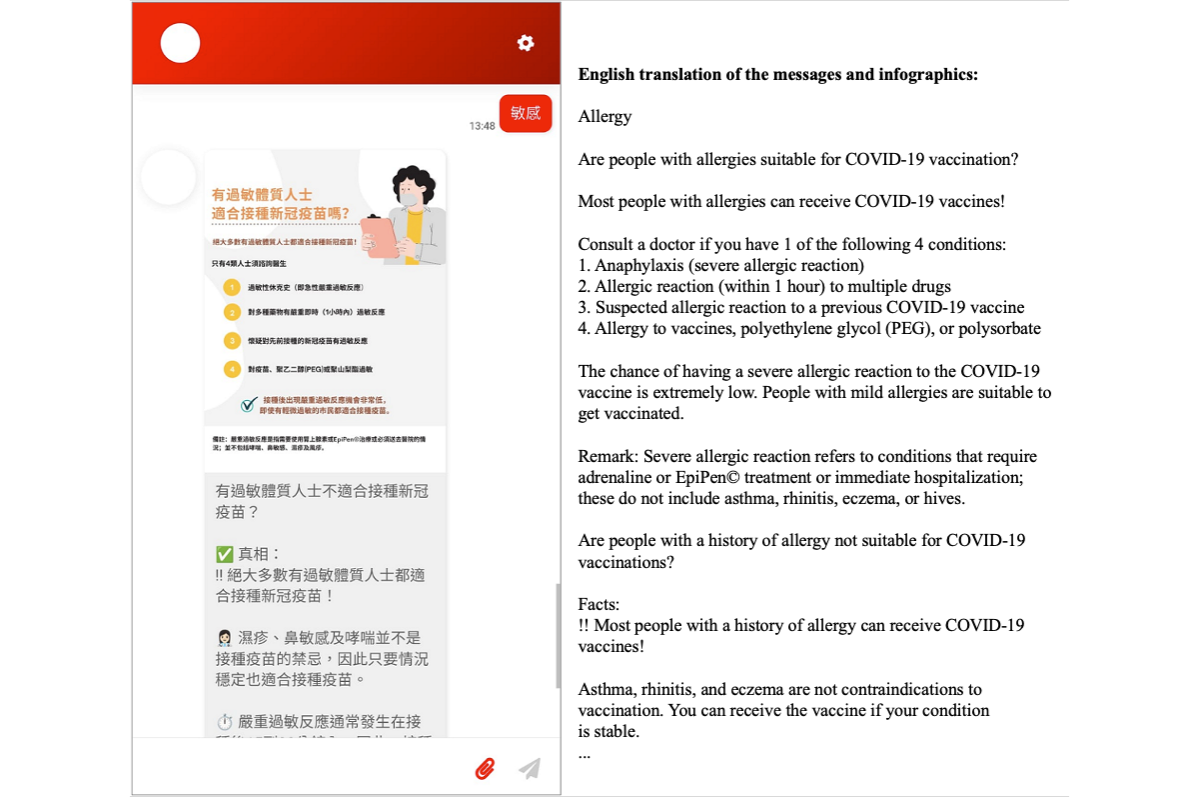
Screenshot of “Vac Chat, Fact Check” showing chatbot navigation by keyword.

The “Vac Chat, Fact Check” chatbot was developed by our team. To promote dissemination, the chatbot could be accessed by any internet browser on smartphones, tablets, and personal computers (ie, web-based). The chatbot was available in Chinese only since most Hong Kong residents (>90%) spoke Chinese. Upon entering the chatbot, the user received a message about how to use the chatbot and a menu of options showing the core functionality of the chatbot. The users could navigate the chatbot by typing the corresponding number of the options in the menus or keywords (eg, allergy) to obtain information directly (Figures 2 and 3).

The chatbot conversation generally unfolded by following predefined rules or decision trees. To better simulate human interactions, the chatbot also used natural language processing (NLP) powered by Google Dialogflow for handling small talk (eg, greetings and thank-yous). The chatbot provided responses mainly using texts with emojis, but some messages also included infographics (Figure 3).

The intervention content followed the Confidence, Complacency and Convenience (“3C’s”) model of vaccine hesitancy [19]. Specifically, the information addressed the lack of trust in the effectiveness and safety of the vaccine (confidence), the lack of perceived risk of COVID-19 or the perception that vaccination is not necessary (complacency), and barriers to access the vaccine (convenience). The information provided by the chatbot was categorized into 6 major topics (Table 1). Our population-based survey suggested that inadequate knowledge about COVID-19 could contribute to vaccine hesitancy [16]. Therefore, the chatbot included general information about COVID-19. Since 2 types of vaccine (inactivated and mRNA vaccines) were available in Hong Kong with differing eligibility criteria (age and pregnancy status), the chatbot also included a decision aid for selecting the suitable vaccine.

**Table 1:** Overview of the topics and contents of “Vac Chat, Fact Check.”

Topic	Content
Information about COVID-19	Symptoms and complications, including “long COVID” Route of transmission and incubation period High-risk populations
Information about COVID-19 vaccination	Mechanism of the vaccines Vaccine efficacy Possible side effects Eligibility for vaccination
Reasons for getting vaccinated	Protection of self Protection of others
Myths and facts about the COVID-19 vaccine	Alleged side effects (eg, infertility and miscarriage) Safety of the vaccine (eg, alteration of a person’s DNA) Safety in people with preexisting conditions (eg, a history of allergy) Lack of efficacy
Information about COVID-19 vaccine boosters	Eligibility for receiving boosters Reasons for receiving boosters
Information about how to get vaccinated	Government’s web-based booking system Venues for vaccination

### Instruments

#### COVID-19 Vaccine–Related Outcomes

All COVID-19 vaccination outcomes were measured at preintervention and postintervention. COVID-19 vaccination status was assessed by asking “have you been vaccinated against COVID-19?” with responses options of “yes, 2 doses,” “yes, 1 dose,” and “no.” Intention to receive the COVID-19 vaccine (for those responded “no”) or COVID-19 boosters (for those responded “yes, 2 doses”) were assessed on a scale from 1 (not likely at all) to 5 (very likely) [20]. By adapting an item from the OCEANS study [21], we also asked, “if people around you were thinking of getting a COVID-19 vaccination, you would...” Responses were coded from 1 (suggest that they do not get the vaccination) to 5 (strongly encourage them).

The main efficacy outcome was changes in COVID-19 vaccine hesitancy from preintervention to postintervention. We adapted the Vaccine Hesitancy Scale (VHS) developed by the WHO’s Strategic Advisory Group of Experts on Immunization for assessing COVID-19 vaccine hesitancy [22]. The COVID-19 VHS included 9 Likert-style items, each coded from 1 (strongly disagree) to 5 (strongly agree; Multimedia Appendix 1 [22,23]). After the reverse coding of some items, all items were summed to give a total score ranging from 9 to 45, with higher scores indicating greater COVID-19 vaccine hesitancy. The VHS can also be divided into the “Lack of confidence” subscale (7 items) and the “Risk” (2 items) subscale and analyzed.

In our sample, the COVID-19 VHS had high internal consistency preintervention (Cronbach *α*=.86) and postintervention (Cronbach *α*=.88) [24]. Concurrent validity was supported by a higher mean VHS score in unvaccinated participants versus those who received 1 dose and 2 doses of vaccine (28.6 vs 26.4 vs 23.0, respectively; *P*<.001). The VHS score was also inversely and moderately correlated to intention to receive the vaccine (Spearman *ρ*=–0.48; *P*=.01) or boosters (Spearman *ρ*=–0.55; *P*<.001) and willingness to encourage others to vaccinate (Spearman *ρ*=–0.64; *P*<.001) [25].

In the 4-month follow-up, we assessed the COVID-19 vaccination status in initially unvaccinated participants by their responses of “no” and “yes, [number] dose(s).” For booster-hesitant participants, we asked whether they had received a booster shot (“yes” or “no”).

#### Usability and Acceptability Outcomes

The postintervention questionnaire included the System Usability Scale (SUS), a widely used instrument in software engineering, to measure the participants’ perceived usability of the chatbot. The 10-item SUS gives a composite score ranging from 0 to 100, with 68 or above indicating above-average usability [26]. Other acceptability measures included the perceived usefulness of the chatbot in (1) getting information about the COVID-19 vaccine, (2) making decisions about vaccination, and (3) increasing the motivation to get vaccinated, each assessed on a scale from 1 (not useful at all) to 5 (very useful). Overall satisfaction with the chatbot was assessed by asking “how likely would you recommend the chatbot to other people” on a 11-point scale from 0 (not likely at all) to 10 (very likely).

#### Other Measures

The baseline questionnaire included the eHealth Literacy Scale (eHEALS) [27], which has been translated into Chinese and used in our study population [28]. The scale included 8 items, which are summed to give an overall score from 8 to 40. Higher scores indicate greater perceived ability to use health technologies. The eHEALS had high internal consistency in our sample (Cronbach *α*=.91). To assess the perceived susceptibility to and severity of COVID-19, we also asked, “how likely do you think you will contract COVID-19 in the future?” and “how dangerous do you think COVID-19 is to health?” respectively, each with 11-point response options. Data on sociodemographic characteristics, chronic disease, and previous flu vaccination were also collected.

### Statistical Analysis

To evaluate the chatbot efficacy, we used 1-sample, 2-tailed *t* test and Wilcoxon sign-rank test to examine the change in intention to receive a vaccine or booster and COVID-19 VHS scores from preintervention to postintervention. We also examined changes in the “Lack of confidence” and “Risk” subscales of the COVID-19 VHS. The effect size of the pre-post difference in COVID-19 VHS scores (Cohen *d*) was calculated as a mean difference divided by the SD of the mean difference. Our sample size of 46 participants could detect a moderate effect size of 0.43 (Cohen *d*) in the pre-post difference in COVID-19 VHS scores with 80% power at 2-sided 5% level of significance. The corresponding effect sizes detectable were 0.64 for intention to vaccinate (n=21) and 0.60 for intention to receive boosters (n=24). Intervention usability and acceptability were reported descriptively. For the secondary aim, we used bivariate and multivariable linear regression to examine the factors associated with the COVID-19 VHS score in all survey respondents. Factors examined included sociodemographic characteristics, chronic disease status, previous flu vaccination, eHealth literacy, and the perceived susceptibility to and severity of COVID-19.

All statistical analyses were conducted in Stata/MP software (version 15.1; StataCorp). We used complete case analyses because there were no missing data in the web-based survey and postintervention assessment except eHealth literacy (n=2) and the perceived susceptibility (n=4) and severity (n=4) of COVID-19. A 2-sided *P*<.05 denoted statistical significance.

## Results

### Participant Characteristics

The mean (SD) age of all survey respondents was 21.4 (6.3) years, and 61% (177/290) of respondents were female (Table 2). Participants of the pre-post study (n=46) had similar characteristics to those of nonparticipants (n=244) except, as expected, having significantly higher COVID-19 vaccine hesitancy (*P*<.001).

**Table 2:** Characteristics of all survey respondents (N=290).

Characteristic			Survey respondents (N=290)	Included in the pre-post study				*P* value^a^
				No (n=244)		Yes (n=46)		
**Age (years)**								
	Mean (SD)	21.4 (6.4)		21.7 (6.8)	20.2 (2.7)		.15	
	Median (IQR)	20 (19-21)		20 (19-21)	20 (18-21)		.14	
**Sex, n (%)**								
	Male	113 (39)		97 (39.8)	16 (35)		.53	
	Female	177 (61)		147 (60.2)	30 (65)			
**Chronic disease, n (%)**								
	No	270 (93.1)		229 (93.9)	41 (89)		.25	
	Yes	20 (6.9)		15 (6.1)	5 (11)			
**Previous flu vaccination, n (%)**								
	No	156 (53.8)		134 (54.9)	22 (48)		.38	
	Yes	134 (46.2)		110 (45.1)	24 (52)			
**eHealth literacy^b^**								
	Mean (SD)	30.2 (4.6)		30.2 (4.7)	30.1 (4.0)		.86	
	Median (IQR)	32 (28-32)		32 (28-32)	31.5 (28-32)		.56	
**Perceived susceptibility to COVID-19^c^**								
	Mean (SD)	3.2 (1.9)		3.2 (1.9)	3.2 (1.6)		.94	
	Median (IQR)	3 (2-5)		3 (2-5)	3 (2-5)		.68	
**Perceived severity of COVID-19^c^**								
	Mean (SD)	6.3 (2.2)		6.3 (2.2)	6.5 (2.2)		.58	
	Median (IQR)	7 (5-8)		7 (5-8)	7 (5-8)		.61	
**COVID-19 vaccine hesitancy^d^**								
	Mean (SD)	23.6 (5.8)		22.6 (5.3)	28.6 (5.6)		<.001	
	Median (IQR)	23 (20-27)		23 (19-26)	29 (23-33)		<.001	

**^a^**Calculated by chi-squared test, 2-sample, 2-tailed *t* test, or Wilcoxon rank-sum test as appropriate.

^b^Assessed by the eHealth Literacy Scale; possible scores range from 8 to 40, with higher scores indicating greater eHealth literacy.

^c^Assessed by an 11-point scale from 0 to 10; higher scores indicate greater perceived susceptibility or severity.

^d^Assessed by the COVID-19 Vaccine Hesitancy Scale; possible scores range from 9 to 45, with higher scores indicating greater vaccine hesitancy.

### Factors Associated With COVID-19 Vaccine Hesitancy

In all survey respondents, both bivariate and multivariable models showed that lower eHealth literacy and perceived danger of COVID-19 were associated with higher COVID-19 vaccine hesitancy (Table 3). The results were similar after additionally adjusting for COVID-19 vaccination status (data not shown).

**Table 3:** Factors associated with COVID-19 vaccine hesitancy^a^ (N=290).

Factor	Crude B (95% CI)	*P* value	Adjusted B (95% CI)^b^	*P* value
Age (years)	0.038 (–0.068 to 0.14)	.48	–0.031 (–0.15 to 0.086)	.60
Sex, female	1.16 (–0.21 to 2.53)	.10	0.79 (–0.061 to 2.18)	.27
Had chronic disease	1.70 (–0.94 to 4.34)	.21	1.65 (–1.24 to 4.54)	.26
Had previous flu vaccination	–1.65 (–5.21 to 1.90)	.35	–0.040 (–1.38 to 1.30)	.95
eHealth literacy^c^	–0.27 (–0.42 to –0.13)	<.001	–0.26 (–0.41 to –0.11)	<.001
Perceived susceptibility to COVID-19^d^	0.17 (–0.20 to 0.54)	.36	0.20 (–0.17 to 0.57)	.29
Perceived severity of COVID-19^d^	–0.35 (–0.66 to –0.050)	.02	–0.41 (–0.71 to –0.10)	.009

^a^Assessed by the COVID-19 Vaccine Hesitancy Scale; possible scores range from 9 to 45, with higher scores indicating greater vaccine hesitancy.

^b^Adjusting for other variables in the table.

^c^Assessed by the eHealth Literacy Scale; possible scores range from 8 to 40, with higher scores indicating greater eHealth literacy.

^d^Assessed by an 11-point scale from 0 to 10; higher scores indicate greater perceived susceptibility or severity.

### Pre-Post Evaluation of the Chatbot

The completion rate of the postintervention assessment was 100% (46/46). Table 4 shows the favorable changes in all measures related to COVID-19 vaccination from preintervention to postintervention (mean duration: 15.0 days). The main efficacy outcome of COVID-19 VHS score significantly decreased from 28.6 (preintervention) to 24.5 (postintervention), with a mean difference of –4.2 (*P*<.001) and an effect size (Cohen *d*) of 0.94. Similarly, both the “Lack of confidence” and “Risk” subscale scores significantly decreased. Intention to vaccinate or receive boosters and willingness to encourage others to vaccinate significantly increased from preintervention to postintervention. One unvaccinated participant at preintervention reported having received the first dose of the vaccine at postintervention.

**Table 4:** Changes in COVID-19 vaccine-related measures from preintervention to postintervention (n=46).

		Preintervention	Postintervention	*P* value^a^
**COVID-19 vaccine hesitancy (n=46)^b^**				
	Mean (SD)	28.6 (5.6)	24.5 (6.0)	<.001
	Median (IQR)	29 (23-33)	25 (20-29)	<.001
**COVID-19 vaccine hesitancy: Lack of confidence (n=46)^c^**				
	Mean (SD)	20.8 (5.0)	17.2 (5.2)	<.001
	Median (IQR)	21 (16-26)	18 (13-21)	<.001
**COVID-19 vaccine hesitancy: Risk (n=46)^d^**				
	Mean (SD)	7.8 (1.3)	7.2 (1.6)	.01
	Median (IQR)	8 (7-8)	7.5 (6-8)	.02
**Intention to vaccinate (n=21)^e^**				
	Mean (SD)	3.0 (0.73)	3.9 (0.83)	<.001
	Median (IQR)	3 (3-4)	4 (3-4)	.001
**Intention to receive boosters (n=24)^e^**				
	Mean (SD)	1.9 (0.3)	2.8 (0.9)	<.001
	Median (IQR)	2 (2-2)	3 (2-3)	<.001
**Willingness to encourage others to vaccinate (n=46)^f^**				
	Mean (SD)	2.7 (1.0)	3.0 (0.9)	.04
	Median (IQR)	3 (2-3)	3 (2-4)	.04

^a^Calculated by paired 2-tailed *t* test or Wilcoxon signed-rank test as appropriate.

^b^Assessed by the COVID-19 Vaccine Hesitancy Scale (VHS); possible scores range from 9 to 45, with higher scores indicating greater vaccine hesitancy.

^c^“Lack of confidence” subscale of the COVID-19 VHS; possible scores range from 7 to 35, with higher scores indicating greater lack of confidence in the vaccine.

^d^“Risk” subscale of the COVID-19 VHS; possible scores range from 2 to 10, with higher scores indicating greater perceived risk of the vaccine.

^e^Assessed on a scale from 1 (not likely at all) to 5 (very likely).

^f^Assessed on a scale from 1 (suggest that they do not get the vaccination) to 5 (strongly encourage them).

### Usability and Acceptability of the Chatbot

On average, the participants used the chatbot for a total of 64 (SD 47) minutes during the 1-week trial period. Longer time spent on the chatbot was correlated with a larger reduction in vaccine hesitancy with marginal significance (Spearman *ρ*=0.26; *P*=.08). Among participants who used the chatbot (n=46), the median (IQR) SUS score was 72.5 (65-77.5) out of 100. On a scale from 1 (not agree at all) to 5 (strongly agree), the median (IQR) score on the perceived usefulness of the chatbot was 4 (4-4) for getting information about the COVID-19 vaccine, 3 (2-4) for making decisions about vaccination, and 3 (2-3) for increasing the motivation to get vaccinated. The median (IQR) recommendation score was 7 (6-8) on a scale from 0 to 10.

### Vaccination Status at 4-Month Follow-up

Overall, 18 (82%) of the 22 initially unvaccinated participants and 19 (79%) of the 24 booster-hesitant participants responded to the post hoc 4-month follow-up. All 18 unvaccinated participants reported having received COVID-19 vaccination (2 doses: n=16, 89%; and 1 dose: n=2, 11%), whereas 7 (37%) of the 19 booster-hesitant participants reported having received boosters.

## Discussion

### Principal Findings

This pilot study showed a significant decrease in COVID-19 vaccine hesitancy after using the “Vac Chat, Fact Check” chatbot in young adults who were hesitant to vaccinate or receive boosters. According to the rule of thumb of Cohen [29], the effect size (Cohen *d*=0.94) was large. Other efficacy outcomes, including intention to vaccinate or receive boosters and willingness to encourage others to vaccinate, consistently showed the benefit of the chatbot. The usability of the chatbot was supported by the median SUS score of 72.5 out of 100, which fell between the cutoffs of “good” (a score of 71.4) and “excellent” (a score of 85.5) adjective ratings [30]. The median recommendation score of 7 on a scale from 0 to 10 indicated the satisfactory acceptability of the chatbot [31].

Our PubMed search using the keywords of vaccine and chatbot and their synonyms only identified 1 peer-reviewed study that provided empirical evidence on the efficacy of a chatbot for promoting COVID-19 vaccination. The study was a web-based experiment on a French sample population, which found that interacting with a chatbot could promote more positive attitudes toward COVID-19 vaccines and intention to vaccinate [32]. A study (preprint) also showed an increase in vaccine acceptance in Japanese adults after using “Corowa-kun,” a chatbot in LINE instant messenger [33]. Direct comparison between our study with these studies were difficult because of differences in the study methods, sample characteristics, and outcome measures. Nevertheless, our findings were consistent with these studies by showing a positive impact of chatbot on COVID-19 vaccine uptake.

To our knowledge, our study was the first to include actual receipt of COVID-19 vaccines or boosters as outcome measures in chatbot evaluation. Assuming (conservatively) that all participants lost to follow-up did not receive any vaccine or booster, 82% (18/22) of the initially unvaccinated participants received at least 1 dose of vaccine, whereas 29% (7/24) of booster-hesitant participants received a booster. As a reference, the corresponding rates were 92% and 28% in Hong Kong residents aged 20 to 29 years on March 30, 2022 (same date as the follow-up) [34]. Note that these figures could not be directly compared because of differences in sample characteristics, and our participants were likely more vaccine- or booster-hesitant than the general population. Nonetheless, the satisfactory response rate of 80% (37/46) provides support for the feasibility of conducting longer-term (>3 months) follow-up in future trials.

Corroborating our previous findings in the general population [16], we found that higher perceived severity of, but not susceptibility to, COVID-19 was associated with lower COVID-19 vaccine hesitancy. Previously studies have found that eHealth literacy was associated with knowledge and adherence to nonpharmacological preventive measures against COVID-19 [17,35]. This study further found eHealth literacy to be associated with COVID-19 vaccine hesitancy. Higher eHealth literacy helps people process and discern the credibility of web-based health information, which may buffer the impact of the infodemic (an overabundance of information, both accurate or otherwise, during a disease outbreak) and misinformation against the vaccine and thus hesitancy. Our findings corroborate the importance of building eHealth literacy to fight the COVID-19 pandemic.

Similar to most chatbots built to support health care amid the COVID-19 pandemic [11,12], our “Vac Chat, Fact Check” chatbot was primarily rule-based. We decided against building a chatbot that is entirely powered by NLP for practical reasons. First, existing and readily available NLP engines remain inadequate in handling free-flow conversations in Cantonese (the local Chinese dialect). Second, rule-based chatbots are relatively inexpensive and could be quickly developed and deployed to mitigate the pandemic when health care resources are stretched. NLP-based chatbots could better simulate human interaction but require extensive training and resources to become adequately usable. Nonetheless, our study has provided proof-of-concept evidence to support chatbots as a mode of delivery to promote vaccination, which provides the impetus for developing more sophisticated and potentially more effective chatbots.

### Limitations

The main limitation of the pre-post study is the lack of a control or comparison group, which limited the causal inference of any changes observed after using the chatbot. The possibility that the observed changes were attributable to contextual changes along the course of the outbreak could not be excluded. However, the study was conducted at a time when Hong Kong had been maintaining a low level of local transmission with nearly 0 daily local case (from June to December 2021). This setting, coupled with the short interval between preintervention and postintervention assessments, was unlikely to have had a substantial effect on the vaccination outcomes. Nevertheless, the findings must be considered preliminary and hypothesis-generating. Another limitation is the small sample size, which precludes the examination of the chatbot’s efficacy in sociodemographic subgroups (eg, sex). Third, since all measures were self-reported, social desirability bias could not be excluded. Finally, our study targeted young adults given their greater vaccine hesitancy than older populations and their frequent use of social networking sites—a major source of misinformation. The generalizability of the findings to other populations is unclear. Due to the convenience sampling method, our participants may not be representative of all young adults who are unvaccinated or booster-hesitant.

### Conclusions

Promoting the uptake of COVID-19 vaccines is crucial to mitigating the impact of COVID-19. This pilot study provided initial evidence to support the efficacy, usability, and acceptability of a chatbot for promoting COVID-19 vaccination in young adults who were unvaccinated or booster-hesitant. Randomized controlled trials are warranted to test the effectiveness of the chatbot in increasing COVID-19 vaccination. Although our study indicated the benefits of the chatbot in both unvaccinated and booster-hesitant young adults, the drivers for vaccine hesitancy between the 2 groups likely differ. Further research is also needed to understand their differences to provide more tailored information and optimize the chatbot’s efficacy.

## Appendix

Multimedia Appendix 1 COVID-19 Vaccine Hesitancy Scale.

## Abbreviations

eHEALS: eHealth Literacy Scale
NLP: natural language processing
SUS: System Usability Scale
VHS: Vaccine Hesitancy Scale
WHO: World Health Organization
