# Supplementary material for: Efficacy, Usability, and Acceptability of a Chatbot for Promoting COVID-19 Vaccination in Unvaccinated or Booster-Hesitant Young Adults: Pre-Post Pilot Study
Source: J Med Internet Res. 2022 Oct 4;24(10):e39063. doi: 10.2196/39063 (PMC9534274; doi:10.2196/39063)
Supplement: Multimedia Appendix 1 [file jmir_v24i10e39063_app1.pdf]

Multimedia Appendix. COVID-19 Vaccine Hesitancy Scale

新冠疫苗猶豫量表

您有多同意以下有關接種新冠疫苗的說法？

(5=非常不同意; 4=不同意; 3=一半半; 2=同意; 1=非常同意)

a. 新冠疫苗對我的健康是重要的

b. 新冠疫苗是有效的

c. 接種新冠疫苗對保護社區內其他人是重要的

d. 政府新冠疫苗接種計劃所提供的新冠疫苗都是有益的

e. 新的疫苗比舊有的疫苗帶來的風險高*

f. 我從新冠疫苗接種計劃獲得有關新冠疫苗的資訊都是可靠和可信的

g. 接種新冠疫苗是保護自己免於 2019 冠狀病毒病的一個好方法

h. 一般情況下，我會聽從醫生或醫護人員給我有關新冠疫苗的建議

i. 我擔心新冠疫苗帶來的嚴重副作用*

*反向題

COVID-19 Vaccine Hesitancy Scale

How much do you agree with the each of the following statement on COVID-19 vaccinations?

(5=strongly disagree; 4=disagree;3=neither agree nor disagree;2=agree; 1=strongly agree)

a. COVID-19 Vaccines are important for my health
b. COVID-19 Vaccines are effective
c. Being vaccinated is important for the health of others in my community
d. All COVID-19 vaccinations recommended by the government are beneficial
e. New vaccines carry more risks than older vaccines*
f. The information I receive about COVID-19 vaccines from the COVID-19 Vaccination Programme is reliable and trustworthy
g. Getting COVID-19 vaccines is a good way to protect me from COVID-19
h. Generally, I do what my doctor or healthcare provider recommends about COVID-19 vaccines for me.
i. I am concerned about serious adverse effects of COVID-19 vaccines*

*reversed items

Adapted from the Vaccine Hesitancy Scale developed by the World Health Organization’s SAGE Working Group on Vaccine Hesitancy:

Larson HJ, Jarrett C, Schulz WS, Chaudhuri M, Zhou Y, Dube E, Schuster M, MacDonald NE, Wilson R; SAGE Working Group on Vaccine Hesitancy. Measuring vaccine hesitancy: The development of a survey tool. Vaccine. 2015 Aug 14;33(34):4165-75.

Shapiro GK, Tatar O, Dube E, Amsel R, Knauper B, Naz A, Perez S, Rosberger Z. The vaccine hesitancy scale: Psychometric properties and validation. Vaccine. 2018 Jan 29;36(5):660-667.
